# Visually grounded emotion regulation via diffusion models and user-driven reappraisal

**DOI:** 10.3389/frai.2026.1691445

**Published:** 2026-03-04

**Authors:** Edoardo Pinzuti, Oliver Tüscher, André Ferreira Castro

**Affiliations:** 1 Leibniz Institute for Resilience Research, Mainz, Germany; 2 Department of Psychiatry, Psychotherapy and Psychosomatic Medicine, University Medical Center Halle, Halle (Saale), Germany; 3 German Center for Mental Health (DZPG), Site Halle-Jena-Magdeburg, Halle (Saale), Germany; 4 Department of Psychiatry and Psychotherapy, University Medical Center of the Johannes Gutenberg-University Mainz, Mainz, Germany; 5 School of Life Sciences, Technical University of Munich, Freising, Germany

**Keywords:** affective computing, affective responses, AI-generated visual feedback, aversive images, cognitive emotion regulation (CER) task, cognitive reappraisal, diffusion models, emotion regulation

## Abstract

Cognitive reappraisal is a key strategy in emotion regulation, involving reinterpretation of emotionally charged stimuli to alter affective responses. Despite its central role in clinical and cognitive science, real-world reappraisal interventions remain cognitively demanding, abstract, and primarily verbal in nature. This reliance on higher-order cognitive and linguistic processes can be especially impaired in individuals with trauma, depression, or dissociative symptoms, limiting the effectiveness of standard approaches. Here, we propose a novel, visually based augmentation of cognitive reappraisal by integrating large-scale text-to-image diffusion models into the emotional regulation process. Specifically, we introduce a system wherein users reinterpret emotionally negative images via spoken reappraisals, which are then transformed into supportive, emotionally congruent visualizations using stable diffusion models with a fine-tuned IP-adapter module. This generative transformation visually instantiates users' cognitive reappraisals while maintaining structural similarity to the original stimuli, thus externalizing and reinforcing regulatory intent. To evaluate this approach, we conducted a within-subjects experiment (*N* = 20) using a modified cognitive emotion regulation (CER) task. Participants reappraised or described aversive images from the international affective picture system (IAPS), with or without AI-generated visual feedback. Results indicate that AI-assisted reappraisal significantly reduced negative affect relative to both non-AI reappraisal and control conditions. Further analyses show that sentiment alignment between participant reappraisals and generated images correlates with affective relief, suggesting that multimodal coherence enhances regulatory efficacy. Our findings highlight the feasibility of using generative visual support for cognitive reappraisal. This work opens a new interdisciplinary direction at the intersection of generative AI, affective computing, and therapeutic technology design.

## 1 Introduction

Emotion regulation is a central process in human psychological functioning, underpinning a wide range of outcomes related to mental health, interpersonal behavior, and adaptive decision-making (Gross, 2002; Lincoln et al., 2022; Gross, 2015). Within this domain, cognitive reappraisal—defined as the reinterpretation of an emotional stimulus to modify its impact—has emerged as a particularly effective strategy (Hajcak et al., 2010; Riepenhausen et al., 2022). Empirical research has consistently demonstrated that reappraisal can reduce negative affect, lower physiological arousal, and serve as a protective factor against stress-related disorders across both clinical and non-clinical populations (Han et al., 2023; In et al., 2021; Southward et al., 2022). It is also a cornerstone of cognitive-behavioral therapy (CBT), supported by a robust body of experimental and neuroimaging data (Bryant et al., 2021; Buhle et al., 2014; Halfmann et al., 2021). These findings have contributed to its widespread use in both laboratory paradigms and therapeutic interventions, underscoring its relevance as a tool for promoting emotional resilience (McRae et al., 2012; Montfort and Sondheim, 2024).

Standardized laboratory procedures, such as the Cognitive Emotion Regulation (CER) task, have provided key insights into the mechanisms of reappraisal (Kanske et al., 2011). In such paradigms, participants are typically instructed to mentally reinterpret negatively valenced stimuli—often drawn from the International Affective Picture System [IAPS, (Lang et al., 1997)]—in ways that diminish their emotional salience (Erk et al., 2010; Sandner et al., 2021). Functional neuroimaging studies employing these tasks have shown that successful reappraisal is associated with reduced amygdala activity (Banks et al., 2007; Klumpp et al., 2017) and enhanced activation in prefrontal regions implicated in cognitive control (Ray and Zald, 2012; Morawetz et al., 2016). However, despite its empirical validity, cognitive reappraisal remains a cognitively demanding and introspective technique that hinges on abstract verbal reasoning (Vy et al., 2024; Sass et al., 2013; McRae, 2016). This requirement places significant demands on executive function, which can limit the strategy's effectiveness in real-world contexts, particularly during emotionally intense episodes or among individuals with cognitive impairments or those suffering from mental health conditions such as depression, anxiety disorders and post-traumatic stress disorder (PTSD) (Gross and Muñoz, 1995; Kraaij and Garnefski, 2019; Jacob and Anto, 2016; Lincoln et al., 2022).

Given these constraints, there is increasing interest in augmenting reappraisal with computational tools that can reduce cognitive load and facilitate more accessible emotion regulation (Bucci et al., 2019; Mitsea et al., 2023). Despite the growing interest in applying artificial intelligence (AI) to mental health support, the integration of generative AI, such as, large language models (LLMs), into emotion regulation interventions remains nascent (Dehbozorgi et al., 2025; Miner et al., 2017; Rubin et al., 2025; Pataranutaporn et al., 2023; Glickman and Sharot, 2025; Sharma et al., 2023). Recent developments in conversational agents powered by LLMs have enabled more fluent and empathetic interactions, with some studies demonstrating the capacity of LLMs to generate high-quality cognitive reappraisals (Zhan et al., 2024; Chiu et al., 2024). However, these systems are largely limited to passive, text-based outputs that do not actively engage users in multimodal processing (Prochaska et al., 2021; Vaidyam et al., 2019).

Meanwhile, advances in text-to-image generation–particularly through latent diffusion models such as stable diffusion–have enabled efficient and high-quality synthesis of photorealistic and semantically rich images from textual prompts (Blattmann et al., 2023; Zhang et al., 2023; Podell et al., 2023). More recently, image-conditioned variants like IP-Adapter architectures have extended this capability by allowing models to receive both reference images and guiding text, preserving visual structure while modifying semantic or emotional content (Ye et al., 2023a). Early explorations into AI-generated imagery for emotional reflection have shown promise in enhancing affective engagement, yet they remain disconnected from explicit reappraisal strategies and lack systematic evaluation of their emotional impact (Yang et al., 2024; Lomas et al., 2024). Despite their expressive potential, these models have not yet been applied to affective computing or emotion regulation due potential societal representational biases in those models (Luccioni et al., 2023). Existing applications have largely focused on aesthetic style transfer, avatar personalization, or facial expression synthesis, without grounding the visual output in user-driven cognitive reinterpretation (Mittenentzwei et al., 2024). Thus, the potential of image-conditioned generative models to serve as interactive, affective scaffolds–instantiating user-authored reappraisals in visually recontextualized form–remains unexplored (Ruppert and Eiroa-Orosa, 2018; Yamada et al., 2018; Pearson et al., 2015). The opportunity to leverage these technologies for real-time co-regulation of emotional meaning between human users and AI agents represents a critical, unmet need in both computational psychiatry and human-computer interaction (Bell et al., 2024; Blackwell, 2021).

To address this gap, we introduce a novel framework for visual cognitive reappraisal that integrates generative diffusion models into an emotion regulation task. Participants reinterpret negative images through verbal reappraisal, which is transcribed and used—alongside the original image—to condition a stable diffusion XL model with IP-Adapter. The resulting AI-generated image reflects the user's reinterpretation, offering a visually grounded, emotionally resonant scaffold for regulation. In a controlled study (*N* = 20), we show that this AI-assisted approach significantly reduces negative affect compared to traditional mental-only reappraisal. Furthermore, affective improvement was predicted by sentiment alignment between the reappraisal prompt and the generated image, highlighting the role of multimodal coherence in emotional regulation. Our findings highlight the value of aligning visual output with user intent, and demonstrate how generative AI can act as a real-time cognitive amplifier, bridging abstract thought and emotional transformation. This work opens a new interdisciplinary direction at the intersection of generative AI, affective computing, and therapeutic technology design.

## 2 Methods

### 2.1 Subjects

Twenty healthy adults (10 female, 10 male; age range: 25–55 years, mean = 35.45 ± 8.86) participated in the study. Participants were screened for a history of psychiatric or neurological disorders. Exclusion criteria included current use of psychotropic medication and insufficient fluency in one of the four supported languages (English, Italian, German, French). Participants provided informed consent in accordance with institutional ethical guidelines. Language preference was respected during task interaction: participants could complete the task in their native language or in English if fluent. Three participants opted for English over their native language. Verbal responses were recorded in the preferred language and subsequently translated to English for model input and analysis to align with model training distributions and avoid linguistic bias.

### 2.2 Experimental design

We used a modified Cognitive Emotion Regulation (CER) task (Kanske et al., 2011) to compare traditional and AI-assisted reappraisal. The CER task is a widely adopted paradigm for studying cognitive strategies that alter affective experience (Erk et al., 2010; Sandner et al., 2021). We adapted it to incorporate real-time generative image feedback. Each participant completed trials under one of four within-subject conditions in a 2 × 2 design: Describe, Describe AI, Reappraise, and Reappraise AI.

At the start of each trial, participants viewed a neutral or negative image from the International Affective Picture System [IAPS; (Lang et al., 1997)] for 4 s. This period was intended to allow sufficient time to visually encode the stimulus. Afterward, a microphone icon signaled participants to speak aloud, either by describing the image (Describe conditions) or by reinterpreting it in a more positive light (Reappraise conditions). The original image remained onscreen during this verbalization period to preserve visual context. Spoken input was chosen over typing to reduce distraction and cognitive switching. Participants were given 12 s to speak.

In the AI conditions, verbal utterances were transcribed in real time using OpenAI's Whisper-turbo speech recognition system (Radford et al., 2022). If spoken in a language other than English, transcripts were automatically translated without manual correction. These English transcripts, together with the original image, were passed as input to a Stable Diffusion XL model equipped with an IP-Adapter module (Ye et al., 2023a). To account for image generation time, a gray screen was presented for a duration of 4 s. The resulting image reflected the participant's interpretation and was shown for 3 s. To ensure timing was matched across all conditions, a gray screen was also shown for 4 s after the verbal phase in the non-AI conditions. After this, participants rated their emotional state using a visual analog scale ranging from very positive to very negative. The rating interface consisted of a horizontal slider positioned below five emoticon anchors representing discrete levels of valence. The slider was mapped to integer values from 1 to 9. Subsequently, for visualization and analysis, we remapped affective rating scale such that 0 indicated a neutral response, with values ranging from −2 (strongly negative) to +2 (strongly positive). Participants were trained on how to interpret each emoticon prior to beginning the task.

Training also included examples for both verbal conditions. In the Reappraise conditions, participants were instructed to create an alternative, positive narrative or outcome for the scene (e.g., “This person will recover,” or “They are being rescued”). Describe trials served as a low-level verbal baseline, allowing comparisons that isolate the contribution of reinterpretation and/or generative visual augmentation.

### 2.3 Subject speech handling

Participants were allowed to respond in their native languages to reduce cognitive and emotional interference during the reappraisal task. Prior research indicates that using a non-native language can increase working-memory load and reduce emotional resonance, thereby impairing regulation efficacy (Pavlenko, 2012; Caldwell-Harris, 2015). Speech was transcribed using chat GPT Whisper, a multilingual ASR model trained on over 680,000 h of audio, which achieves near–human-level accuracy across major languages (Radford et al., 2022). Translations were performed using Google's Neural Machine Translation system (Wu et al., 2016) and manually screened for semantic consistency. No major transcription or translation errors were identified. Illustrative examples of the automatic speech recognition → translation → image generation pipeline have been added to Supplementary Figure S1 to demonstrate typical quality and transformation consistency.

A 12-s speaking window was implemented to balance cognitive feasibility with the latency constraints of real-time text-to-image generation. Prior studies on emotion-regulation tasks typically use 6–10-s intervals for silent or written reappraisals (Goldin et al., 2008; Denny et al., 2009). Pilot testing confirmed that participants could comfortably articulate full vocal reappraisals within 10–12 s; extending this window would have increased verbal working-memory demands and inter-individual variability, potentially diminishing regulation effectiveness (Sheppes et al., 2015; Kalisch, 2009). To verify that this constraint did not bias outcomes, we report the distribution of trial durations and the percentage of trials completed within 12 s (Supplementary Figure S2), which shows that nearly all reappraisals fell comfortably within the time limit. Finally, to rule out potential time-related confounds, we examined correlations between speaking duration and ratings across all conditions in Supplementary Figure S7; no significant relationships were observed, indicating that speaking time did not systematically influence reported affect.

To assess whether the translation process introduced systematic language-specific effects, we further examined both the distribution of sentiment scores and their relationship with affective ratings across source languages. Mean sentiment scores were highly comparable across languages (German: *M* = 0.065, *SD* = 0.628; English: *M* = 0.068, *SD* = 0.607; French: *M* = 0.108, *SD* = 0.663; Italian: *M* = 0.064, *SD* = 0.621), indicating no substantial language-dependent shift in sentiment magnitude following translation. We then computed trial-level correlations between sentiment scores and affective ratings separately for each language, pooling all trials within language. The association was positive and consistent across languages (German: *r*≈0.52; English: *r*≈0.39; French: *r*≈0.78; Italian: *r*≈0.63), demonstrating stable relationships between sentiment and affective outcome across linguistic groups. Notably, the French estimate reflects data from a single participant and should be interpreted with caution. Together, these analyses suggest that the translation process did not introduce systematic bias in sentiment scoring or in its relationship to affective outcomes, supporting the generalizability of the observed effects across source languages.

### 2.4 Linear mixed-effects modeling

Linear mixed-effects models (LMMs) were used to analyze affective ratings while accounting for both subject-level and stimulus-level variability. This approach allows simultaneous estimation of fixed effects (experimental factors) and random effects (subjects and stimuli), providing a more accurate representation of the hierarchical structure of the data. Models were fitted in R using the lme4 package.

The first model examined the effects of image type and task condition on affective ratings:


 
  m1  < - lmer(
    rating ~ image_type * condition +
      (1 | subject) + (1 | stimulus)
    data = df
    REML = TRUE
  )
 


Here, *image_type* (negative vs. neutral) and *condition* (Reappraisal, Reappraisal-AI, View, View-AI) were included as fixed effects, and random intercepts were specified for *subject* and *stimulus* (IAPS image).

To further evaluate the influence of semantic and affective features of participants' reappraisals, a second model incorporated *Alignment Score* and *Sentiment Score* as continuous predictors. Alignment (z-scored) was modeled as a moderator interacting with image type and condition, while sentiment (z-scored) was included as an additive covariate controlling for general affective tone:


 
  m2  < - lmer(
    rating ~ image_type * condition * alignment_z
    + sentiment_z +
      (1 | subject) + (1 | stimulus)
    data = df
    REML = TRUE
  )
 


Estimated fixed effects, standard errors, and 95% confidence intervals were extracted using the lmerTest package.

### 2.5 Stable diffusion model XL

Stable Diffusion XL (SDXL) is a high-capacity text-to-image generative model that extends the latent diffusion framework introduced in prior work (Podell et al., 2023). Rather than generating images directly in pixel space, SDXL operates in a compressed latent space defined by a pretrained variational autoencoder (VAE), enabling efficient sampling while preserving high-resolution image quality. The core generative process is implemented via a U-Net denoising architecture, conditioned on text embeddings produced by a frozen CLIP-based encoder (Che et al., 2023). This encoder converts natural language prompts into a sequence of token representations, which are injected into the U-Net through a series of cross-attention layers, guiding image synthesis at multiple spatial scales. Compared to earlier iterations of Stable Diffusion, SDXL introduces a dual-text encoder configuration and supports high-fidelity generation at resolutions up to 1, 024 × 1, 024 pixels. These improvements yield more robust prompt interpretability, as well as finer control over stylistic and semantic content.

### 2.6 IP-adapter for visual conditioning

To enable joint conditioning on both visual and textual information, we incorporate the IP-Adapter architecture proposed by (Ye et al., 2023a), adapting it to function within a frozen SDXL backbone. The IP-Adapter is a lightweight plug-in module (approximately 22 million parameters) designed to inject reference image features into the diffusion process via a decoupled cross-attention mechanism, without modifying the pre-trained model weights.

A frozen CLIP ViT-H/14 encoder is used to extract a global embedding from a reference image (Radford et al., 2021). This embedding is projected through a small trainable network–comprising a linear layer followed by LayerNorm–into a fixed-length sequence of image tokens (see Section 2.7). These tokens share the same dimensionality as the text embeddings from SDXL's CLIP-based text encoder, allowing direct integration into the U-Net's attention layers.

In each cross-attention block of the SDXL U-Net, the IP-Adapter introduces a parallel attention stream dedicated to image-based features. The query projection matrix *W*^*Q*^ is shared between modalities, while separate key and value projections (*W*^*K′*^, *W*^*V′*^) are learned for the image pathway. The text-based and image-based outputs, denoted *O*_*t*_ and *O*_*i*_ respectively, are initially combined via simple addition:


$$\begin{array}{l} O_{\text{combined}}=O_{t}+O_{i} \end{array}$$ (1)

To allow for finer control over the contribution of the image features, we introduce a conditioning scale parameter λ∈[0, 1]:


$$\begin{array}{l} O_{\text{combined}}=O_{t}+\lambda \cdot O_{i} \end{array}$$ (2)

Here, λ modulates the relative influence of visual guidance. A lower value of λ emphasizes the text prompt, while higher values strengthen the impact of the image features. We adopt values in the range λ∈[0.3, 0.7] consistent with prior work (Ye et al., 2023a), tuning it to balance semantic coherence and visual fidelity.

This approach enables visual grounding while preserving SDXL's pre-trained text-to-image generation capabilities. Because all original model weights (including the U-Net and text encoder) remain frozen, the adapter imposes minimal overhead and supports efficient fine-tuning. Only the projection layers for image embeddings are updated during training. The decoupled attention structure ensures that visual and textual signals are integrated without interference, which is particularly valuable in emotionally grounded tasks such as cognitive reappraisal. Here, the goal is to transform affective meaning while retaining the spatial structure and visual context of the original image'a balance that the IP-Adapter is well-suited to achieve.

### 2.7 Dataset construction for stable diffusion IP-adapter

To adapt the IP-Adapter for emotion-related imagery, we constructed a synthetic dataset of image–prompt pairs representing realistic affective reinterpretations rather than training it on behavioral or emotional labels. Starting from 160 base images (80 negative, 80 neutral) drawn from IAPS (Lang et al., 1997), we used GPT-3.5 (OpenAI, 2023a; Ye et al., 2023b) to generate ten reappraisal prompts per image. Where available, few-shot prompting incorporated actual human reappraisals from pilot subjects to anchor generation in realistic tone and structure. Each prompt was paired with its corresponding base image and passed to Stable Diffusion XL (SDXL 1.0) (Podell et al., 2023) for generation, using the IAPS image as a visual reference through the image prompt pathway.

To enhance semantic and aesthetic fidelity, we performed post-generation verification and ranking using GPT-4 Vision (OpenAI, 2023b). For each image, GPT-4V was prompted to evaluate whether the output visually matched the intended emotional transformation described in the prompt. Images flagged as semantically inconsistent, emotionally incoherent, or containing visual artifacts were discarded. The retained images were further augmented using standard pixel-level transformations, including symmetry, cropping, noise injection, and brightness jitter, to generate ten additional variants per sample, yielding over 16, 000 total image–prompt pairs. Additionally, 20% of prompts were paraphrased using ChatGPT-3.5 (OpenAI, 2023a) with synonym replacement strategies to introduce lexical variety and reduce overfitting.

After automated and manual quality control, including artifact filtering and sentiment consistency checks, less than 8% of samples were removed. The resulting dataset comprised 18, 000 high-quality image–prompt pairs, each reflecting a reappraised interpretation of an emotionally neutral or negative scene. This dataset was used to fine-tune the IP-Adapter to improve text–image alignment and semantic fidelity for reappraisal-related prompts, while keeping all SDXL weights frozen. The adapter's objective was to enhance general visual–semantic alignment in emotion-related content rather than to learn cognitive or affective representations. All prompts used in this dataset were generated directly from Whisper-transcribed human speech (Radford et al., 2022), and with no grammatical corrections applied, preserving natural variation and spoken syntax.

We note that the same set of IAPS images was used as visual anchors for constructing the synthetic training dataset and as experimental stimuli during behavioral testing. However, the IP-Adapter was trained solely on these images paired with automatically generated text prompts, without access to any human affective ratings, task labels, or behavioral responses. The adapter's objective was to learn a generic visual–text alignment function to improve semantic fidelity of reappraisal imagery, not to model emotional responses or behavioral outcomes. All SDXL weights remained frozen during training, preventing the model from memorizing image–label associations. While this partial overlap means the adapter was exposed to the same base visual content, it did not receive any supervision related to the experimental outcomes. We acknowledge this as a limitation in terms of full data independence and have clarified this in the manuscript and response letter.

### 2.8 IP-adapter fine-tuning and semantic guidance scaling

We trained the IP-Adapter module on a dataset of 18,000 paired images and captions, using the same denoising objective employed by the base diffusion model. During training, all weights of the underlying SDXL model were frozen; only the parameters of the IP-Adapter were updated. To improve generalization and enable flexible conditioning at inference, we applied classifier-free guidance dropout (Ho and Salimans, 2022) randomly omitting the image embedding, the text embedding, or both with low probability. This strategy encourages the model to operate robustly under partial or missing conditioning signals. Training was conducted over 200,000 optimization steps using the AdamW optimizer (learning rate: 1e-4) with a batch size of 48, distributed across four NVIDIA L40 GPUs (48GB VRAM each). Total training time was approximately 54 h. Representative comparisons between outputs from the pre-trained and fine-tuned models are shown here. Due to copyright restrictions, they cannot be displayed in this paper. These comparisons illustrate the motivation behind training the IP-Adapter module. The original Stable Diffusion model, when used without fine-tuning, frequently produced anatomically implausible outputs–such as phantom limbs, distorted body parts, or objects unnaturally misaligned with human figures. Fine-tuning the IP-Adapter substantially improved the structural coherence and semantic fidelity of the generated images.

At inference, the pipeline accepts both a text prompt *p* and a reference image *I* (or just an image if no prompt is provided). The CLIP image encoder and projection head generate a sequence of image tokens *F*_*i*_, while the SDXL text encoder produces a sequence of text embeddings *F*_*t*_ corresponding to *p*. The U-Net denoiser, augmented with parallel cross-attention mechanisms, synthesizes an image from noise over *T* denoising steps, conditioned jointly on *F*_*t*_ and *F*_*i*_. We use the DDIM sampler (Song et al., 2020) with a text guidance scale of 7.5.

The relative influence of the visual reference can be modulated by scaling the image-based attention component *O*_*i*_ with a factor α before it is combined with the text-based attention output *O*_*t*_. This allows precise control over the generative balance between textual and visual information. For the Reappraisal-AI condition, α was dynamically adapted for each image to account for variability in semantic content across IAPS stimuli. While some images depict complex, structured scenes, others are sparse or ambiguous (e.g., involving injury or destruction), making semantic reinterpretation more difficult.

In the Describe-AI condition, α was fixed at 0.8 to ensure that the generated images remained closely aligned with the visual content of the original image while still integrating information from the descriptive prompt. This produced consistent generations that preserved core visual features of the input stimulus.

### 2.9 Sentiment analysis of verbal reappraisals and predictive validity for affective ratings

To evaluate the affective tone of participants' verbal responses during the task, we performed sentiment analysis directly on the subject prompts – the verbal content produced by participants in response to each image transcribed as text to input the diffusion model. We used the the multilingual RoBERTa-based model (Barbieri et al., 2020) to obtain probabilities for three sentiment labels: neutral, negative and positive. The sentiment score was computed as a continuous polarity index ranging from −1 (clearly negative) to +1 (clearly positive), incorporating the model's full label probability distribution. Each probability was weighted according to its position on the sentiment scale: −1 for *negative*, 0 for *neutral*, and +1 for *positive*. Formally:


$$\begin{array}{l} \text{Sentiment Score}=P\left(\text{negative}\right)\times \left(-1\right)+P\left(\text{neutral}\right)\times 0\\ \;+P\left(\text{positive}\right)\times \left(+1\right) \end{array}$$

This results in a continuous sentiment measure that reflects both the direction and strength of affective content in the subject's prompt. This score ranges from −1 (clearly negative) to +1 (clearly positive), capturing the overall emotional polarity of the subject's verbal response. We conducted a Pearson correlation analysis (ρ) to assess the relationship between the sentiment of participants' verbal reappraisals and their self-reported affect ratings, stratified by condition and image valence. To ensure that the observed associations were not confounded by prompt length or linguistic complexity, we repeated the analysis using linear regression while controlling for word count. We report these results in (Figure S4).

To ensure that alignment estimates were not dependent on the GPT-4V captioning pipeline, we additionally computed an objective CLIP-based cross-modal similarity metric, defined as the cosine similarity between the participant's reappraisal text embedding and the corresponding image embedding in CLIP space. This alternative alignment score was analyzed using the same mixed-effects framework and is reported in Supplementary Figure S8.

### 2.10 Semantic alignment between verbal reappraisal and corresponding generated imagery

To assess the semantic alignment between participants' reappraisal prompts and the content of the corresponding AI-generated images, we employed a two-step pipeline combining image captioning and embedding-based similarity analysis. For each trial in the *Reappraisal AI* condition, the reappraised image was presented to GPT-4V (via the OpenAI API) along with the standardized instruction: “*This image was generated as a positive reinterpretation of a scene. Describe what this new image communicates emotionally and semantically.”* GPT-4V returned a free-text caption describing both the scene and its emotional message. In cases where GPT-4V declined to generate a caption–typically due to safety filters or low salience–we used a fallback captioning pipeline based on a CLIP image encoder paired with a language decoder to generate a descriptive caption.

To quantify semantic consistency, we embedded both the participant's verbal reappraisal prompt (*reappraisal prompt*) and the AI-generated image caption (*image caption*) using the all-mpnet-base-v2 model from the sentence-transformers library. Each text was converted into a fixed-length embedding:


$$\begin{array}{lll} {\text{r}}_{i} & = & \text{encode(reappraisal prompt)}\\ {\text{c}}_{i} & = & \text{encode(image caption)} \end{array}$$

Semantic alignment was then computed as the cosine similarity between the two vectors:


$$\begin{array}{l} {\text{Alignment}}_{i}=\cos\left(\theta \right)=\frac{{\text{r}}_{i}\cdot {\text{c}}_{i}}{\left||{\text{r}}_{i}||||{\text{c}}_{i}|\right|} \end{array}$$

Higher values indicate stronger semantic congruence between the participant's intended reinterpretation and the emotional content inferred from the generated image. This continuous alignment metric was used in downstream correlation analyses to evaluate whether greater prompt–image coherence was predictive of reductions in negative affect and increases in perceived emotional congruence.

## 3 Results

### 3.1 Stable Diffusion-generated images maintain structural fidelity in reappraisal prompting

To systematically evaluate the effectiveness and translational potential of generative AI for cognitive emotion regulation (CER), we designed a controlled CER task based on Kanske et al. (2011) incorporating Stable Diffusion XL (SDXL) augmented with IP-Adapter conditioning (Ye et al., 2023a). Participants were exposed to emotionally aversive stimuli drawn from IAPS, followed by a verbal reappraisal phase in which they cognitively reappraised the presented content toward a more neutral or positive interpretation. These verbal reappraisals were transcribed in real time and repurposed as conditioning prompts for the SDXL model, which generated semantically aligned visual reinterpretations grounded in both the original image and the user-supplied cognitive transformation.

**Figure 1:**
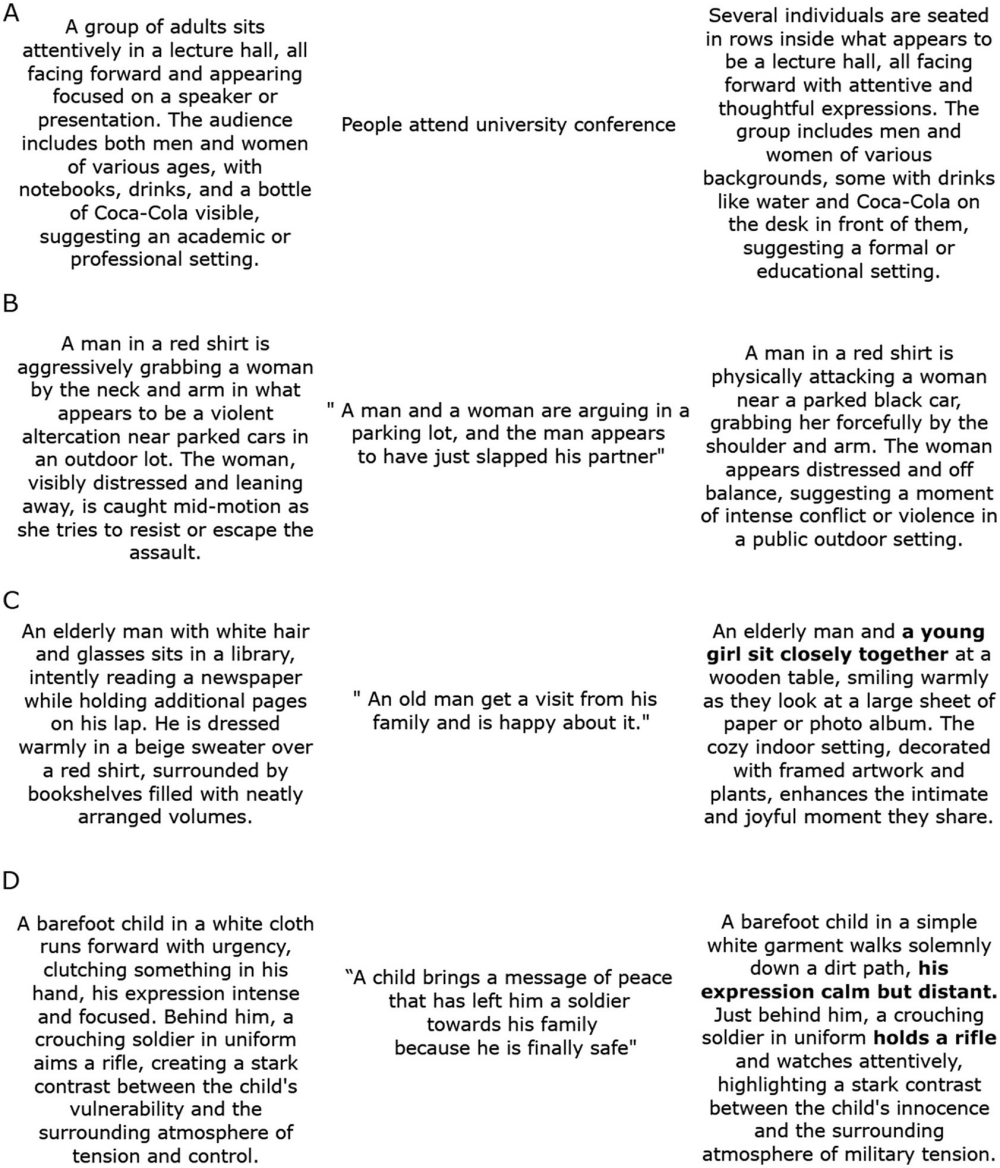
Examples of reappraised image pairs generated using Stable Diffusion. Representative examples of image transformations across experimental conditions, generated using the Stable Diffusion XL model with IP-Adapter conditioning. Each triplet shows the original IAPS stimulus (left), the transcribed verbal prompt (center), and the resulting AI-generated image (right). Prompts were captured in real time via OpenAI's Whisper-turbo voice-to-text system and passed into the generation pipeline without manual correction, preserving participants' spontaneous speech, including minor grammatical or typographic errors. Because both the original IAPS images and the Stable Diffusion outputs derived from them cannot be displayed due to copyright restrictions, their visual content is instead represented here through textual descriptions generated by ChatGPT. Bold text in the verbal prompt indicates the participant's reappraisal content, highlighting the linguistic segment that guided the emotional reinterpretation of the scene. Original IAPS stimuli and corresponding Stable Diffusion images were shown to editors and reviewers, and can be seen here. **(A, B)** illustrate outputs from the *Describe-AI (neutral)* and *Describe-AI (negative)* conditions, respectively. In these cases, image transformations primarily reflected the participant's verbal descriptions of visual features from the original stimulus, preserving the original affective tone. **(C, D)** show results from the *Reappraisal-AI (neutral)* and *Reappraisal-AI (negative)* conditions.

Figure 1 presents representative outputs from the generative pipeline, illustrating the effectiveness of the SDXL + IP-Adapter architecture in visually instantiating participant-generated reappraisals. Across conditions, the generated images maintained high structural fidelity to the original IAPS inputs while integrating semantic and affective cues derived from participants' verbal prompts. In the *Describe-AI (neutral)* and *Describe-AI (negative)* conditions, one class of transformations involved the generation of neutral or negative image features as described by the participant based on IAPS images (Figures 1A, B). In contrast, the *Reappraisal-AI (neutral)* and *Reappraisal-AI (negative)* conditions yielded image transformations that reflected positive reappraisals of originally neutral scenes, or the attenuation or removal of aversive content, respectively (Figures 1C, D).

It is important to note that a subset of outputs exhibited subtle artifacts or semantically diffuse renderings. These limitations are consistent with known challenges of diffusion-based models in affect-sensitive contexts, motivating further refinement of multimodal conditioning fidelity and control (Blattmann et al., 2023; Zhang et al., 2023; Podell et al., 2023).

### 3.2 Stable diffusion-generated images modulate emotional responses in cognitive reappraisal task

We formally tested two hypotheses regarding the affective modulation induced by AI-augmented cognitive reappraisal. First, we hypothesized that combining cognitive reappraisal with generative visual feedback (Reappraisal AI; RAI) would significantly reduce negative affect ratings relative to traditional reappraisal without imagery (Reappraisal; R). Second, we posited that the mere inclusion of AI-generated imagery in the absence of reappraisal (Describe AI; DAI) would not significantly modulate affective ratings–either for negative or neutral stimuli–when compared to a passive description baseline (Describe), thus isolating the regulatory contribution of reappraisal semantics from the presence of imagery alone (see Section 2).

As a preliminary validation of our CER paradigm, we examined the overall sensitivity of participants' affective ratings to experimental manipulation across all conditions (*N* = 20). Specifically, we compared mean emotional responses to negative and neutral images across conditions. As expected, reappraised negative images elicited significantly higher affective ratings (*M* = −0.18, *SD* = 0.68) than non-reappraised negative images (*M* = −1.07, *SD* = 0.48), with a mean difference of 0.88. This was supported by a significant main effect of instruction, *F*(1, 19) = 47.33, *p* < 0.001, and a significant instruction × emotion interaction, *F*(1, 19) = 15.79, *p* = 0.001 (see Table 1). These results confirm that our CER task successfully captures established emotional regulation effects, replicating patterns observed in prior literature and validating the task's construct fidelity.

**Table 1:** Repeated-measures ANOVA results with partial η^2^ effect sizes.

**Index**	**F Value**	**Num DF**	**Den DF**	**Pr > F**	** $\eta_{partial}^{2}$ **
Emotion	116.801100	1.000000	19.000000	0.000000	0.860100
Instruction	47.332100	1.000000	19.000000	0.000000	0.713600
Modality	25.485200	1.000000	19.000000	0.000100	0.572900
Emotion:instruction	15.790800	1.000000	19.000000	0.000800	0.453900
Emotion:modality	5.511100	1.000000	19.000000	0.029900	0.224800
Instruction:modality	28.671600	1.000000	19.000000	0.000000	0.601400
Emotion:instruction:modality	8.074900	1.000000	19.000000	0.010400	0.298200

Repeated-measures ANOVA results with partial η^2^ effect sizes. A 2 (emotion: negative vs. neutral) × 2 (instruction: describe vs. reappraise) × 2 (modality: with AI vs. without AI) repeated-measures ANOVA was conducted to examine the effects of task condition on self-reported emotional valence. The table reports *F*-values, degrees of freedom (DF), and associated *p*-values. All main effects and higher-order interactions were statistically significant. Notably, the three-way interaction (*emotion:instruction:modality*) was significant [*F*(1, 19) = 8.07, *p* = 0.0104], indicating that the effectiveness of AI support varied as a function of both the emotional valence of the stimulus and the type of instruction given. This supports the interpretation that AI-generated imagery enhances reappraisal effectiveness selectively for negatively valenced stimuli.

Specifically, we compared mean emotional responses to negative and neutral images across conditions. As expected, reappraised negative images elicited significantly higher affective ratings (*M* = −0.18, *SD* = 0.68) than non-reappraised negative images (*M* = −1.07, *SD* = 0.48), with a mean difference of 0.88. This was supported by a significant main effect of instruction, *F*(1, 19) = 47.33, *p* < 0.001, and a significant instruction × emotion interaction, *F*(1, 19) = 15.79, *p* = 0.001 (see Table 1). These results confirm that our CER task successfully captures established emotional regulation effects, replicating patterns observed in prior literature and validating the task's construct fidelity. In addition, significant main and interaction effects involving modality (*F*(1, 19) = 25.49, *p* < .001, $\eta_{partial}^{2}=.57$) and a three-way interaction among emotion, instruction, and modality (*F*(1, 19) = 8.07, *p* =.01, $\eta_{partial}^{2}=.30$) indicate that the presence of AI-generated imagery modulated the effectiveness of reappraisal differently across emotional contexts. Notably, the effect sizes for all significant factors were large (all $\eta_{partial}^{2}>0.20$), underscoring the robustness of these effects. These findings corroborate the selective enhancement of regulatory outcomes observed in the Reappraisal AI condition. Nonetheless, we would like to not that these estimates should be interpreted cautiously given the exploratory sample size (*N* = 20).

**Figure 2:**
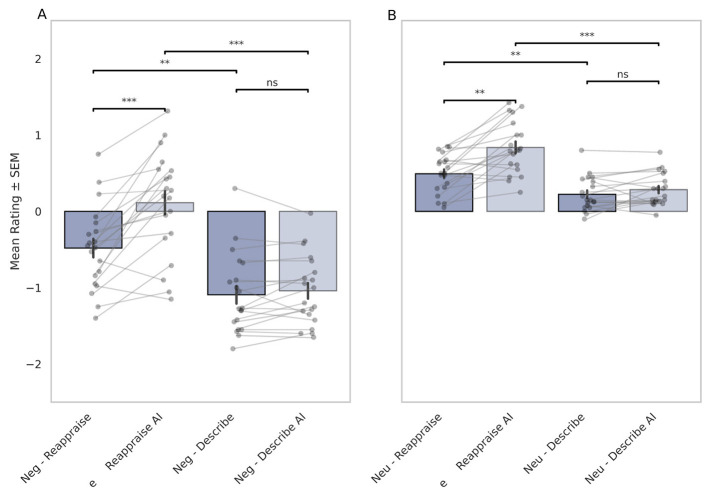
Effect of AI-assisted reappraisal on emotional valence ratings across stimulus types. Mean-centered emotional valence ratings are shown by condition for negative **(A)** and neutral **(B)** images from the IAPS dataset. Each dot represents a participant's mean response per condition; gray lines connect repeated measures within subjects. Statistical significance was assessed via repeated-measures ANOVA with Bonferroni-corrected pairwise comparisons. Significance markers indicate: ***p < 0.001, **p < 0.01, *p < 0.05; “ns” denotes non-significant differences (*p*≥0.05). Error bars represent the standard error of the mean (SEM).

To test the first hypothesis, we compared participants' mean emotional ratings for negative images in the Reappraisal AI (Neg-RAI) and traditional Reappraisal (Neg-R) conditions (*N* = 20). For negative stimuli, participants in the Neg-RAI condition reported significantly reduced negative affect compared to those in the NEG-R condition (Figure 2A; mean difference Neg-RAI vs Neg-R = 0.818, *post-hoc t*-test: *t* = 5.20, *p* < 0.001 (Bonferroni corrected)). Traditional reappraisal alone produced intermediate affective responses, showing improvement relative to passive descriptive viewing (Neg-D) but falling short of the benefit conferred by AI-assisted reappraisal (Figure 2A; mean difference Neg-R vs Neg-D = 0.6, *Post-hoc t*-test: *t* = 4.14, *p* < 0.05, Bonferroni corrected).

Along those lines, affective ratings of negative images in the Reappraisal AI (Neg-RAI) were significantly lower than the descriptive condition with AI-generated imagery (Neg-DAI; mean difference Neg-RAI vs Neg-DAI = 1.15; *post-hoc t*-test: *t* = 7.107, *p* < 0.001, Bonferroni corrected)). Taken together, these results provide strong empirical support for the first hypothesis, underscoring the additive regulatory value of AI-generated visual reinterpretation in amplifying the cognitive mechanisms of reappraisal.

To evaluate the second hypothesis, we assessed whether affective ratings for the descriptive viewing of negative images (Neg-D) differed from those in the descriptive condition with AI-generated imagery (Neg-DAI). As shown in Figure 2A, there was no significant difference between these conditions (mean difference = 0.05, n.s.), indicating that the observed AI-driven effects were specific to the reappraisal of emotionally aversive content rather than to its mere verbal description or visual augmentation.

We further examined whether affective ratings differed between the Neu-D and Neu-DAI conditions, which involved the descriptive viewing of neutral images with and without AI-generated imagery. Consistent with Hypothesis 2, no statistically significant difference was observed between these conditions (mean difference = 0.06, n.s.; Figure 2B). This finding reinforces the interpretation that the presence of AI-generated imagery alone is insufficient to modulate affect in the absence of explicit cognitive reappraisal. These null results support the conclusion that the affective improvements observed in the Neg-RAI condition (negative Reappraisal with AI) are not attributable to visual input alone, but rather to the interaction between semantic reinterpretation and regulatory intent embedded in the reappraisal process.

Interestingly, we also tested whether AI-assisted reappraisal could enhance affective modulation for neutral stimuli (Figure 2B). Here, a significant difference emerged: the neutral Reappraisal AI (Neu-RAI) condition yielded significantly higher (i.e., more positive) ratings than the traditional reappraisal (Neu-R) condition (mean difference = 0.34, *post-hoc t*-test: *t* = 4.77, *p* < 0.01, Bonferroni corrected)). This finding suggests that even in low-arousal contexts, coupling cognitive reinterpretation with personalized generative imagery amplifies the emotional regulatory effect, further supporting the synergistic benefit of combining reappraisal content with visual scaffolding.

Taken together, across both negative and neutral conditions, no statistically significant differences were observed between the Describe AI and Describe-only groups. This reinforces the interpretation that, in the absence of explicit cognitive reappraisal, AI-generated images alone do not meaningfully alter emotional experience (see Figures 2A, B). These findings further underscore the specificity of the cognitive-AI interaction and highlight the necessity of coupling generative output with intentional, user-driven reinterpretation to achieve effective emotion regulation.

### 3.3 Linear mixed-effects modeling confirms robustness of AI-assisted reappraisal effects

**Figure 3:**
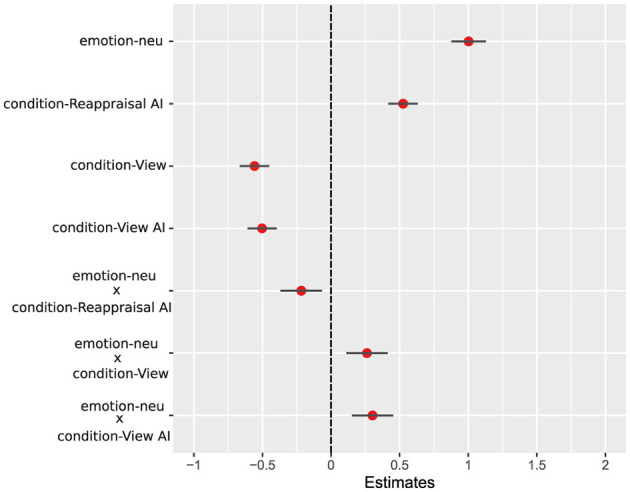
Fixed-effect estimates from linear mixed-effects model. Points (red) represent standardized fixed-effect coefficient estimates (β ) from the linear mixed-effects model, with horizontal lines (black) indicating 95% confidence intervals. Estimates are shown relative to the reference condition, defined as negative images in the Reappraisal condition. Positive coefficients indicate higher affective ratings relative to this reference, whereas negative coefficients indicate lower ratings. Interaction terms reflect deviations from additivity relative to the reference levels. Random intercepts for subject and stimulus were included in the model.

To further verify the robustness of these findings, we conducted an additional linear mixed-effects analysis accounting for both subject- and stimulus-level variability (see Section Methods 2.4). We tested whether AI-assisted reappraisal selectively modulates affective ratings for negative images using a linear mixed-effects model with image type, condition, and their interaction as fixed effects, and random intercepts for subjects and image stimuli (Figure 3).

For negative images, the Reappraisal-AI condition yielded higher (less negative) affective ratings than standard Reappraisal (β = 0.53, SE = 0.05), confirming stronger attenuation of negative affect when reappraisal was supported by AI-generated imagery. Passive viewing conditions (View and View-AI) produced significantly lower ratings relative to Reappraisal, indicating that this effect was specific to active regulation (Figure 3).

The *image type* × *condition* interaction showed that the Reappraisal-AI benefit was reduced for neutral images (β = −0.22, SE = 0.08). Random effects indicated meaningful variability across both subjects and stimuli, demonstrating that the observed effects generalized beyond individual participants and specific IAPS items (Figure 3).

### 3.4 Verbal reappraisal sentiment correlates with affective outcome

**Figure 4:**
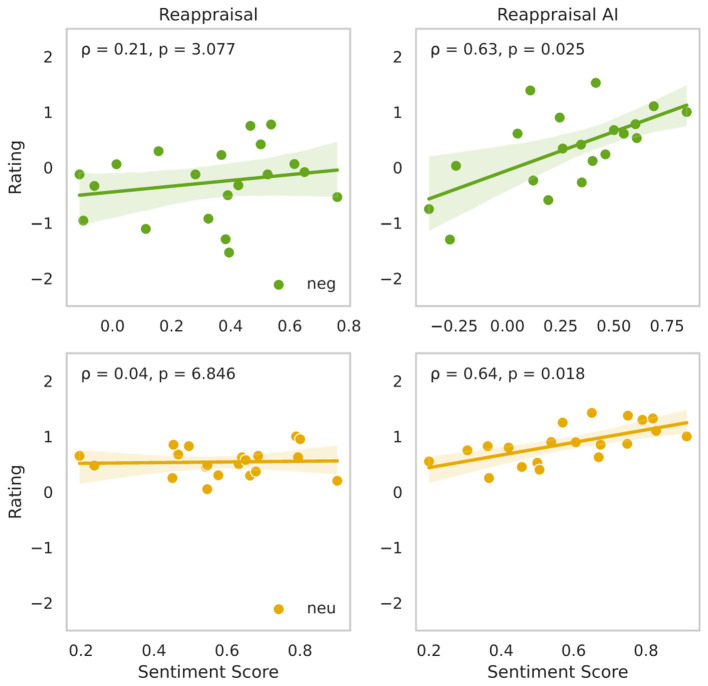
Correlation between sentiment of reappraisal prompts and affective ratings across task conditions. Scatter plots show the relationship between mean sentiment scores (x-axis) and participant-reported affective mean ratings (y-axis) under two task conditions (columns: Reappraisal, Reappraisal AI) and two stimulus types (rows: negative stimuli, top, green; neutral stimuli, bottom yellow). Mean values were computed by averaging across all trials under each condition and stimulus type. Each subplot reports Pearson correlation coefficient (ρ) and the associated two-tailed Bonferroni corrected *p*-value. Shaded areas around regression lines indicate standard error of the mean (SEM), reflecting uncertainty in the fit across subjects.

Next, we wished to investigate the relationship between reappraisal content and emotional outcomes, we analyzed the correlation between sentiment scores extracted from participants' verbal reappraisal prompts and their corresponding affect ratings. Verbal inputs were transcribed using automatic speech-to-text and analyzed using a standard sentiment analysis model, which produced continuous scores reflecting the emotional valence of each utterance, ranging from −1 (strongly negative) to +1 (strongly positive; see Section 2). To evaluate whether the affective tone of participants' verbal prompts correlated with their emotional ratings, we conducted correlations analysis between the sentiment score of each reappraisal or description prompt and the corresponding affective rating, separately for each experimental condition and stimulus valence (Figure 4).

For negative-valence trials, a significant positive correlation was observed in the *Reappraisal-AI (Neg-RAI)* condition (ρ = 0.63, *p* = 0.025), indicating that more positively valenced reappraisal prompts were associated with more favorable (less negative) affective ratings. This relationship was absent in the traditional reappraisal condition (*Neg-R*: ρ = 0.21, *p* = 0.3077), suggesting that the verbal generation of positive reinterpretations alone does not consistently translate into improved emotional outcomes. Instead, these findings support the hypothesis that AI-generated visual imagery plays a critical role in reinforcing the emotional intent of reappraisal content, enabling more effective affective modulation through multimodal alignment.

For neutral-valence trials, a similar pattern emerged. The *Reappraisal-AI (Neu-RAI)* condition showed a statistically significant correlation between prompt sentiment and affective ratings (ρ = 0.64, *p* = 0.018), whereas no such association was observed in the *Reappraisal (Neu-R)* condition (ρ = 0.04, *p* = 6.80.49). This suggests that even in lower-arousal contexts, the presence of visual scaffolding enhances the efficacy of verbal reinterpretations by grounding them in emotionally congruent imagery.

Together, these results underscore the importance of semantic and emotional alignment between participants' reappraisals and the corresponding AI-generated visual outputs (Sharma et al., 2023; Glickman and Sharot, 2025). While sentiment alone is insufficient to drive affective change–as evidenced by the weak or absent correlations in the no-AI conditions–its predictive power emerges when reinforced through personalized generative imagery. These findings support our central hypothesis: that generative AI can serve as an effective amplifier of cognitive reappraisal by transforming abstract regulatory intent into emotionally resonant visual content.

### 3.5 Semantic alignment between reappraisal prompts and generated images predicts affective outcome

To assess whether the effectiveness of AI-assisted reappraisal depends on the degree of semantic congruence between participants' verbal input and the resulting AI-generated imagery, we quantified alignment scores (range from −1, completely dissimilar, to +1, perfectly similar) between each participant's spoken reappraisal prompt and the caption of the corresponding generated image. These captions were derived using a vision-language model (GPT-4V or a fallback CLIP-based captioning model; see Section 2), and both captions and prompts were embedded using a sentence transformer to compute cosine similarity.

**Figure 5:**
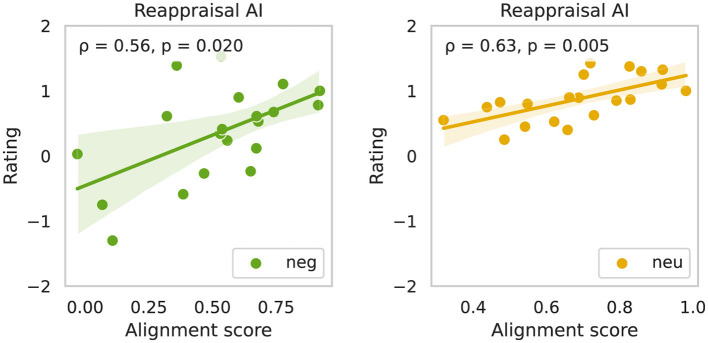
Correlation between image–prompt alignment and affective ratings in the Reappraisal AI condition. Scatter plots show the relationship between alignment scores (x-axis) and participant-reported emotional valence ratings (y-axis) for negative (left, green) and neutral (right, yellow) stimuli, both within the Reappraisal AI condition. Alignment scores quantify the semantic similarity between participant-generated reappraisal prompts and generated captions of the corresponding AI-generated images, computed via cosine similarity of sentence embeddings [using Sentence-BERT (Reimers and Gurevych, 2019)]. Each subplot reports Pearson correlation coefficient (ρ) and associated two-tailed Bonferroni corrected *p*-value. Shaded areas indicate the standard error of the mean (SEM) for the regression fit.

As shown in Figure 5, semantic alignment between the reappraisal prompt and the generated image was significantly correlated with participants' affective ratings. This effect was evident for both negative and neutral image contexts. In the Reappraisal-AI (Neg-RAI) condition, alignment scores were positively associated with less negative ratings (ρ = 0.56, *p* = 0.020), suggesting that the closer the visual output mirrored the user's reappraisal intent, the greater the emotional benefit. Similarly, in the Reappraisal-AI (Neu-RAI) condition, a significant correlation was observed (ρ = 0.63, *p* = 0.005), indicating that even in low-arousal contexts, better alignment enhances the regulatory outcome.

### 3.6 Alignment and sentiment scores independently predict affective ratings in a mixed-effects model

To integrate the semantic and affective predictors examined separately above, A linear mixed-effects model was fitted to affective ratings, including image type (negative vs. neutral), instruction condition (Reappraisal-AI vs. View-AI), and alignment score (z-scored) as interacting predictors, with sentiment score (z-scored) included as an additive covariate. Random intercepts were specified for both subjects and stimuli (Section Methods 2.4).

**Figure 6:**
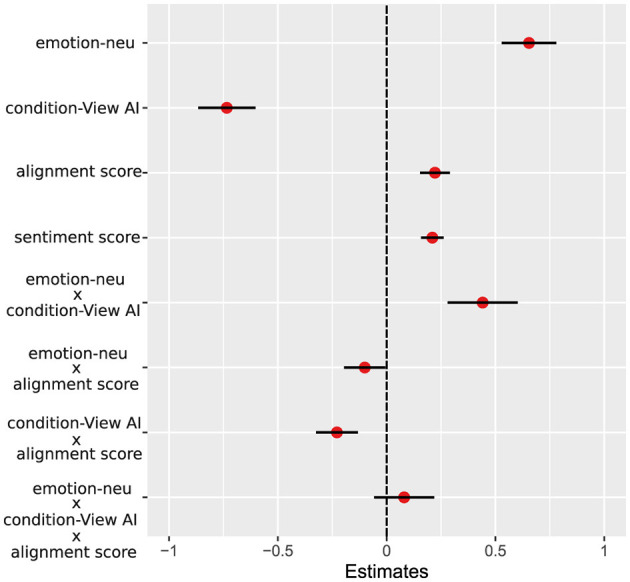
Fixed-effect estimates from the extended mixed-effects model including alignment and sentiment predictors. Points (red) represent standardized fixed-effect coefficient estimates (β) from the mixed-effects model incorporating trial-level alignment and sentiment scores (both z-scored) as continuous predictors. Horizontal lines (black) denote 95 % confidence intervals. Estimates are shown relative to the reference condition, defined as negative images in the Reappraisal-AI condition. Positive coefficients indicate higher affective ratings relative to this reference. Interaction terms represent moderation effects between alignment, image type, and instruction condition. Random intercepts for subject and stimulus were included in the model.

As shown in Figure 6, the model revealed a strong main effect of image type, such that neutral images were rated more positively than negative images (β = 0.65, *t* = 10.21). There was also a robust main effect of instruction condition, with lower affective ratings in the View-AI condition compared to the Reappraisal-AI condition (β = −0.73, *t* = −10.95). Importantly, alignment score positively predicted affective ratings (β = 0.22, *t* = 6.36), indicating that higher alignment between AI-generated content and the intended reappraisal was associated with more positive emotional responses. Sentiment score also showed a significant positive effect (β = 0.21, *t* = 7.95), confirming that general affective tone contributed independently to participants' ratings (Figure 6).

Beyond these main effects, a significant *image type* × *condition* interaction (β = 0.44, *t* = 5.37) indicated that the effect of instruction condition differed between negative and neutral images. Specifically, the reduction in affective ratings observed in the View-AI condition was attenuated for neutral images relative to negative images (Figure 6). Crucially, alignment significantly moderated the effect of instruction condition, as evidenced by a *condition* × *alignment* interaction (β = −0.23, *t* = −4.67). This interaction indicates that the positive association between alignment and affective ratings was stronger in the Reappraisal-AI condition, whereas this relationship was reduced in the View-AI condition.

In addition, a significant *image type* × *alignment* interaction (β = −0.10, *t* = −2.08) suggests that alignment effects were weaker for neutral images than for negative images. The three-way interaction between *image type, condition*, and *alignment* was not significant (β = 0.08, *t* = 1.14), indicating that the moderating role of alignment did not further depend on image valence.

Finally, to validate that these results were not specific to the caption-based alignment metric, we repeated the analysis using a CLIP-based alignment score that directly compares text and image embeddings (Radford et al., 2021). The results were highly consistent (Supplementary Figure S8). These convergent findings demonstrate that the observed effects are robust across two independent formulations of alignment and are not dependent on GPT-4V captioning.

Together, these results indicate that the effectiveness of AI-assisted reappraisal depends on the degree of semantic and emotional alignment between users' reinterpretations and the corresponding AI-generated imagery. Higher alignment and more positive reappraisal tone were each associated with improved affective outcomes, suggesting that multimodal coherence plays a central role in modulating the efficacy of AI-supported emotion regulation.

## 4 Discussion

This study demonstrates that integrating generative AI imagery with cognitive reappraisal significantly enhances affective regulation compared to traditional reappraisal alone. Specifically, participants in the Reappraisal AI (RAI) condition reported more positive emotional outcomes than those in the Reappraisal (R) condition, supporting our first hypothesis. In contrast, emotional ratings in the Describe AI (DAI) condition were indistinguishable from those in the Describe (D) condition across both negative and neutral stimuli, strongly supporting our second hypothesis and reinforcing that the observed affective benefits were driven by reappraisal content rather than the mere presence of AI-generated images. Additionally, sentiment analysis revealed a strong correlation between the positivity of verbal reappraisal prompts and subsequent emotional ratings, while semantic alignment between participant prompts and generated images significantly predicted regulatory success. Together, these findings highlight the potential of generative AI not just as a creative tool, but as a real-time cognitive scaffold capable of augmenting human emotion regulation.

Our findings advance our understanding of cognitive emotion regulation by demonstrating that generative AI can function as an effective augmentative scaffold when tightly coupled with user-driven reappraisal. From a cognitive neuroscience perspective, this work extends existing models of reappraisal by embedding them in a multimodal, interactive loop–bridging internal verbal reframing with externalized visual feedback (Ruppert and Eiroa-Orosa, 2018; Yamada et al., 2018; Pearson et al., 2015). The emotional gains observed in the Reappraisal AI (RAI) condition, relative to traditional reappraisal, suggest that pairing generative models with human cognitive processes can amplify the regulatory effect through richer semantic encoding and reinforcement. Crucially, the absence of affective change in the Describe AI (DAI) condition underscores that the benefits are not attributable to the imagery alone but emerge from the semantic and intentional structure of reappraisal itself.

In the context of affective computing and human-AI collaboration, our results point to a novel use case for generative models: as emotionally aligned, user-conditioned agents capable of participating in shared cognitive tasks (Sharma et al., 2023). Prior systems in this space–such as LLM-based chatbots or unimodal text reappraisal tools–have largely treated emotional support as a conversational or informational problem (Bell et al., 2024; Blackwell, 2021; Prochaska et al., 2021; Vaidyam et al., 2019). In contrast, our approach treats reappraisal as a co-regulated perceptual process, where the AI system visually instantiates the user's intent in real time. This opens pathways toward emotion-aware, interactive systems that respond not just to what users feel, but to how they think about what they feel (Rubin et al., 2025; Glickman and Sharot, 2025).

From a translational perspective, the integration of generative AI into therapeutic contexts such as cognitive behavioral therapy (CBT) represents a promising frontier. Our paradigm demonstrates that personalized, semantically grounded visual feedback can enhance emotional self-regulation in a controlled setting, suggesting potential for deployment in digital mental health platforms (Bucci et al., 2019; Dehbozorgi et al., 2025). Future tools could incorporate generative visual models as adaptive, real-time assistants–augmenting reappraisal strategies, increasing emotional engagement, and lowering cognitive load for users with stress-related or mood disorders (Miner et al., 2017; Lincoln et al., 2022). The paradigm may also hold particular value for populations with limited verbal fluency or executive function, by externalizing abstract thought into concrete visual form (Ruppert and Eiroa-Orosa, 2018; Yamada et al., 2018; Pearson et al., 2015).

### 4.1 Limitations of this study

While the present findings offer compelling evidence for the efficacy of AI-assisted reappraisal, several limitations warrant consideration. First, a key limitation of this study is the relatively small sample size (*N* = 20), which restricts the statistical power to detect subtle behavioral or emotional effects and precludes robust modeling of individual differences or higher-order interactions. This constraint reflects the study's design as a proof-of-concept feasibility investigation, rather than a confirmatory trial. The primary goal was to assess the technical viability and preliminary emotional impact of AI-assisted visual reappraisal, providing an empirical foundation for subsequent large-scale validation. To enhance transparency, we report η^2^ values and include both ANOVA and linear mixed-effects model (LMM) analyses, allowing readers to interpret the magnitude and uncertainty of the observed effects despite the limited *N*. Future work will address this limitation through expanded, powered samples and longitudinal designs that can better capture inter-individual variability in emotional change dynamics.

Second, although sentiment and semantic alignment metrics provided interpretable proxies for affective content, the underlying models (e.g., sentiment analyzers and image captioning systems) are themselves imperfect and may introduce noise or cultural bias (Luccioni et al., 2023). Third, the study relied exclusively on self-reported affect ratings, which–while widely used in affective science–lack the granularity of physiological or behavioral measures (Korpal and Jankowiak, 2018).

In terms of technical constraints, the generative model occasionally produced visually ambiguous or abstract imagery, raising questions about interpretability and user trust in emotionally sensitive contexts. Furthermore, because the IAPS dataset (Lang et al., 1997) was not designed for use with diffusion-based generative models, this may have constrained the semantic fidelity of generated outputs. We also note that the same set of IAPS images were used as visual anchors during IP-Adapter training to construct a synthetic reappraisal dataset. However, no human affective ratings, behavioral data, or task labels were incorporated into the training process, and all SDXL weights remained frozen, preventing any learning of emotional or evaluative patterns. While this partial overlap limits full data independence between the training and experimental phases, the adapter's training objective was restricted to improving text–image alignment fidelity rather than modeling affective responses. We also limited the SDXL inference process to 40 denoising steps to maintain real-time responsiveness, which may have impacted image quality and emotional clarity (Ye et al., 2023a). We also introduced a 4-s silent delay after participants provided their verbal prompt to allow for generation latency. However, this temporal gap may have weakened the immediacy of emotional engagement with the task, potentially reducing the intensity of affective experience and making it harder to detect regulatory effects. Future designs might benefit from tighter stimulus-response coupling or immersive generation environments.

Regarding the safety and ethical guardrails of the diffusion models, this pilot study focused on feasibility rather than safety evaluation, and no explicit filtering mechanisms were implemented to prevent potential 'negative hallucinations'–instances in which the generative model might produce imagery that contradicts or intensifies a user's emotional intent (Aithal et al., 2024; Kim et al., 2024). The semantic fidelity of Stable Diffusion XL and IP-Adapter fine-tuning were relied upon to maintain general coherence between prompts and outputs (Blattmann et al., 2023; Ye et al., 2023a). Nonetheless, the risk of emotionally incongruent or aversive imagery represents a broader challenge for affective computing (Aithal et al., 2024). Future systems should incorporate explicit safety guardrails–such as affective content filters, emotion-consistency classifiers, and participant feedback or opt-out mechanisms–to ensure that multimodal AI systems remain psychologically safe and ethically aligned when applied in mental health or emotionally sensitive contexts (Kim et al., 2024; Oorloff et al., 2025; Pinzuti et al., 2025).

Beyond technical constraints, there are also conceptual and methodological considerations. Although the Describe condition served as a control for verbal processing, prior work suggests that verbal description alone can elicit automatic forms of emotion regulation (Powers and LaBar, 2019; Nook et al., 2022; Lieberman et al., 2007). This may have reduced the contrast between Describe and Reappraisal conditions, potentially underestimating the unique contribution of reappraisal. Nevertheless, we still observed a significant effect of reappraisal with AI support, indicating that the intervention has added value even against this active baseline.

Moreover, while the Describe AI condition was not central to our primary hypotheses, *post-hoc* analyses revealed that alignment and sentiment scores were significantly correlated with affective ratings for negative stimuli (Supplementary Figures S4, S6). On the surface, this may suggest that AI-generated imagery can influence emotional responses even in the absence of explicit reappraisal intent. However, a closer inspection reveals a crucial distinction: although alignment improved affect ratings in Describe AI, participants' emotional responses remained negative on average. In contrast, the Reappraisal AI condition not only showed strong alignment–rating correlations but also produced a consistent shift toward positive affect. This divergence suggests that visual-semantic congruence alone is insufficient for successful emotion regulation unless it is grounded in intentional cognitive reframing. In other words, AI-generated imagery appears to amplify the user's regulatory intent rather than act as a standalone intervention. This interpretation reinforces our central claim that the efficacy of Reappraisal AI is driven not merely by alignment or sentiment, but by the synergistic interaction between generative visual content and reappraisal-oriented verbal input. Nonetheless, future work should more systematically disentangle the unique and interactive effects of alignment, affective tone, and reappraisal intent to clarify the mechanisms underlying these observed benefits.

Finally, our findings reflect short-term emotional modulation within a controlled experimental setting. Future work is needed to evaluate the durability of these effects, their generalizability to naturalistic environments, and the broader ethical implications of using generative models in real-time emotional interventions (Rubin et al., 2025; Pataranutaporn et al., 2023; Sharma et al., 2023; Glickman and Sharot, 2025).

### 4.2 Cognitive mechanisms of visual reappraisal

The observed benefits of AI-assisted visual reappraisal likely arise from a convergence of several well-established cognitive mechanisms. First, externalization–the transformation of internal emotional representations into tangible visual forms–has been shown to facilitate psychological distance and reduce perseverative rumination, enabling individuals to reinterpret affective experiences with greater objectivity (Kross and Ayduk, 2017; Xiong et al., 2022). Second, visualization may reduce working-memory load during reappraisal by offloading part of the cognitive transformation from the internal to the perceptual domain. This effect has been observed in studies linking mental imagery and emotion regulation efficacy (Gross, 2015; Pearson et al., 2015). Finally, visual reappraisal can be understood as a form of attentional redeployment, redirecting cognitive resources away from distress-related imagery toward adaptive reinterpretations (Ochsner and Gross, 2008). Together, these mechanisms provide a theoretical basis for the enhanced emotional impact observed in our study, suggesting that multimodal visualization acts as both a cognitive scaffold and an affective buffer in the reappraisal process. Future research should empirically test these pathways. For example, by combining visual-linguistic reappraisal with eye-tracking, physiological measures (e.g., HRV, EDA), or neural indices of attentional shift, future studies will be able to quantify how externalization and multimodal encoding jointly modulate emotional change.

### 4.3 Conclusion

We presented a multimodal framework that integrates diffusion-based generative models with user-driven cognitive reappraisal to support emotion regulation. By conditioning image generation on verbal reinterpretations of emotionally negative stimuli, the system produces personalized visual outputs that reflect user input while preserving the structural content of the original image. Our results show that AI-assisted reappraisal leads to greater reductions in negative affect compared to traditional reappraisal, and that neither AI-generated imagery nor verbal description alone modulates affective ratings. Further, alignment between user intent and generated image content was predictive of regulatory success, suggesting that semantic consistency plays a critical role in outcome efficacy. While our findings are limited to short-term, self-reported measures, they highlight the feasibility of combining generative models with cognitive scaffolding tasks. Future work should explore how such systems can be extended, personalized, and safely deployed in real-world affective computing and mental health applications.

## Data Availability

The raw data supporting the conclusions of this article will be made available by the authors, upon reasonable request.
