# Supplementary material for: Visually grounded emotion regulation via diffusion models and user-driven reappraisal
Source: Front Artif Intell. 2026 Mar 4;9:1691445. doi: 10.3389/frai.2026.1691445 (PMC12996161; doi:10.3389/frai.2026.1691445)
Supplement: Supplementary file 1 [file Data_Sheet_1.pdf]

6 Supplementary figures

A

An elderly man with white hair and glasses sits in a library, intently reading a newspaper while holding additional pages on his lap. He is dressed warmly in a beige sweater over a red shirt, surrounded by bookshelves filled with neatly arranged volumes.

"Un anziano signore riceve la visita dalla sua famiglia ed è felice per questo."

↓

"An old man get a visit from his family and is happy about it."

An elderly man and **a young girl sit closely together** at a wooden table, smiling warmly as they look at a large sheet of paper or photo album. The cozy indoor setting, decorated with framed artwork and plants, enhances the intimate and joyful moment they share.

B

A barefoot child in a white cloth runs forward with urgency, clutching something in his hand, his expression intense and focused. Behind him, a crouching soldier in uniform aims a rifle, creating a stark contrast between the child's vulnerability and the surrounding atmosphere of tension and control.

"Un bambino porta un messaggio di pace lasciato da un soldato alla sua famiglia perchè finalmente è al sicuro"

↓

"A child brings a message of peace that has left him a soldier towards his family because he is finally safe"

A barefoot child in a simple white garment walks solemnly down a dirt path, **his expression calm but distant.** Just behind him, a crouching soldier in uniform **holds a rifle** and watches attentively, highlighting a stark contrast between the child's innocence and the surrounding atmosphere of military tension.

Figure S1: **Example of translated reappraisal prompts.** The figure illustrates the original Italian text provided by native-speaking participants (top) and the corresponding English translations used for analysis (bottom). Because both the original IAPS images and the Stable Diffusion outputs derived from them cannot be displayed due to copyright restrictions, their visual content is instead represented here through textual descriptions generated by ChatGPT. Bold text in the verbal prompt indicates the participant's reappraisal content, highlighting the linguistic segment that guided the emotional reinterpretation of the scene. Access to the original IAPS stimuli and the corresponding Stable Diffusion images was provided to editors and reviewers and can be seen here.

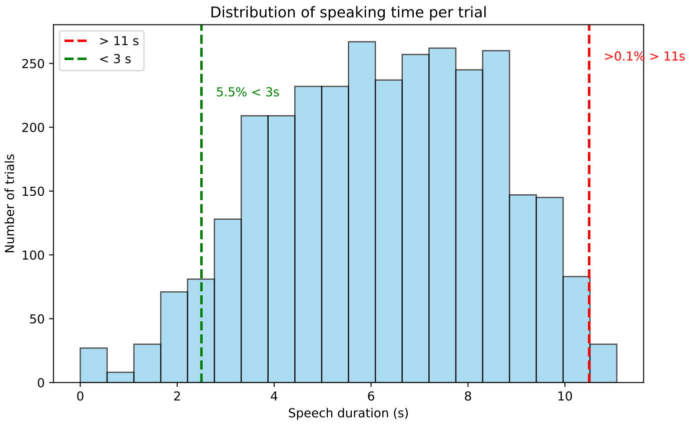

Figure S2: **Distribution of speaking durations across all trials.** The histogram illustrates that the majority of responses clustered between 4–8 seconds, indicating that the 12-second window provided sufficient time for coherent verbal generation without inducing cognitive fatigue or excessive variability.

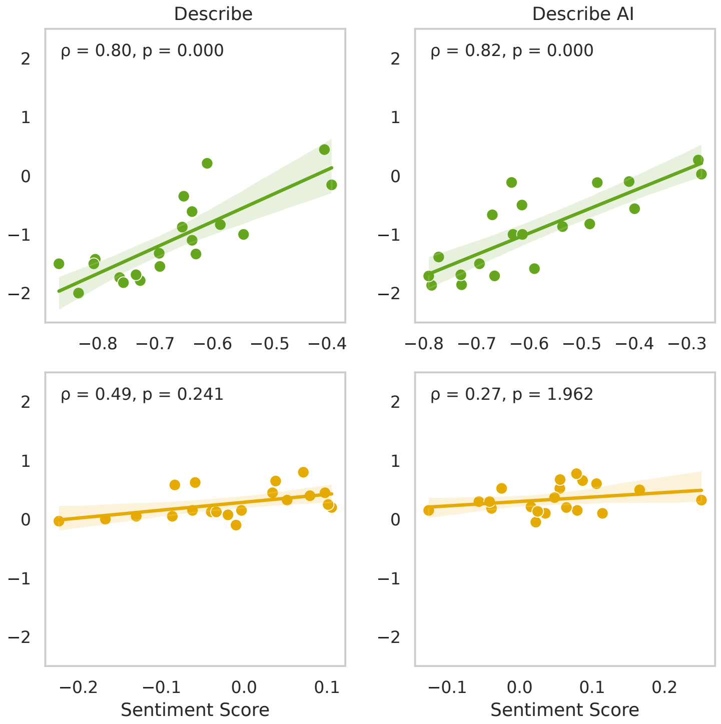

Figure S3: **Correlation between sentiment of reappraisal prompts and affective ratings across Describe and Describe AI conditions.** Scatter plots show the relationship between mean sentiment scores (x-axis) and participant-reported affective mean ratings (y-axis) under two task conditions (columns: Describe, Describe AI) and two stimulus types (rows: negative stimuli, top, green; neutral stimuli, bottom yellow). Mean values were computed by averaging across all trials under each condition and stimulus type. Each subplot reports Pearson correlation coefficient (ρ) and the associated two-tailed p-value. Shaded areas around regression lines indicate standard error of the mean (SEM), reflecting uncertainty in the fit across subjects.

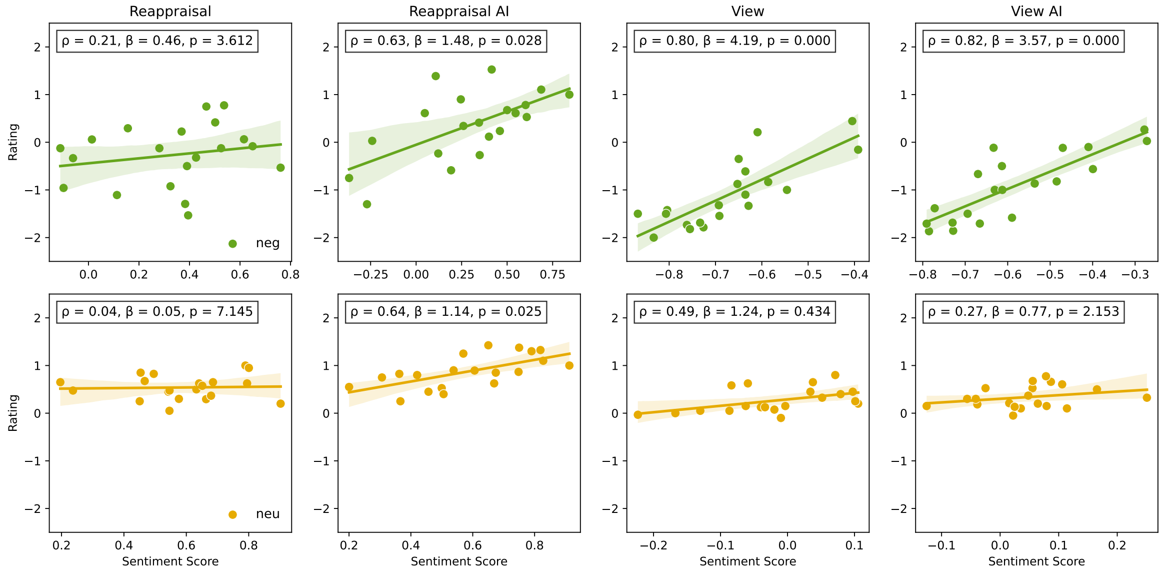

Figure S4: **Regression analyses controlling for linguistic length** The models replicate the analyses shown in Figures 4 and S3, with the addition of word count per trial as a covariate. Including word count did not alter the significance or direction of any effects.

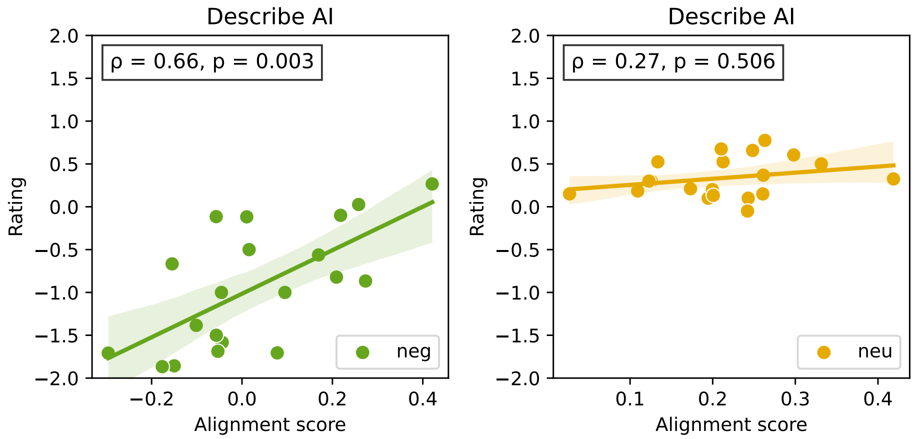

Figure S5: **Correlation between image–prompt alignment and affective ratings in the Describe AI condition**. Scatter plots show the relationship between alignment scores (x-axis) and participant-reported emotional valence ratings (y-axis) for negative (left, green) and neutral (right, yellow) stimuli, both within the Reappraisal AI condition. Alignment scores quantify the semantic similarity between participant-generated reappraisal prompts and generated captions of the corresponding AI-generated images, computed via cosine similarity of sentence embeddings (using Sentence-BERT [69]). Each subplot reports Pearson correlation coefficient (ρ) and associated two-tailed p-value. Shaded areas indicate the standard error of the mean (SEM) for the regression fit.

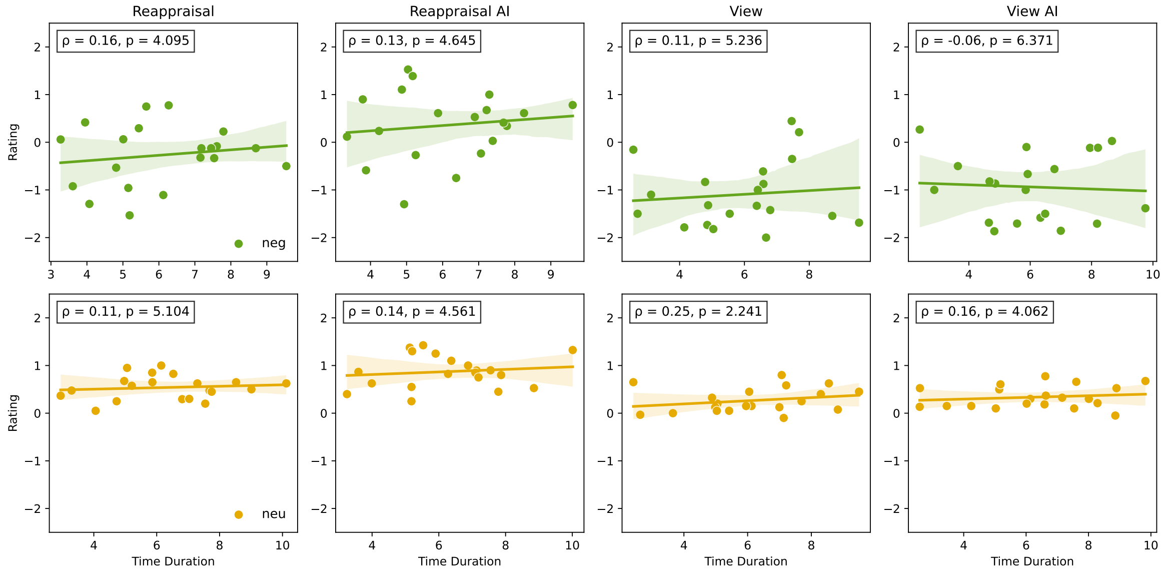

Figure S6: **Correlations between speaking duration and affective ratings across conditions**. Each panel depicts the association between trial duration (x-axis) and rating (y-axis) under Reappraisal, Reappraisal AI, View, and View AI conditions (Color scheme similar to **Figure** 4). Shaded areas represent 95% confidence intervals. No significant correlations were observed in any condition, suggesting that response duration did not affect rating outcomes.

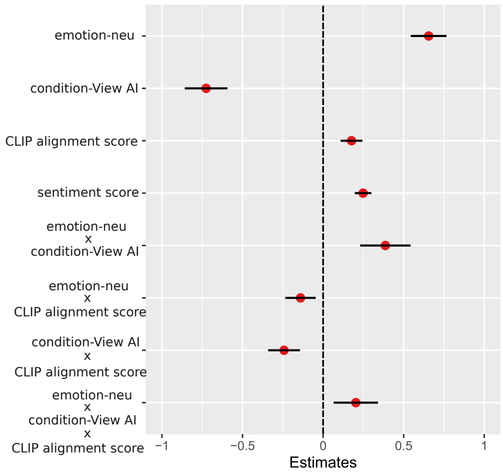

Figure S7: **Fixed-effect estimates from the linear mixed-effects model using the CLIP-based alignment metric.**. Results from the CLIP-based analysis closely replicated the caption-based alignment model reported in the main text. Strong main effects were observed for image type (neutral $>$ negative; $\beta = 0.655$, $t = 11.58$) and condition (View-AI $<$ Reappraisal-AI; $\beta = -0.726$, $t = -10.74$). CLIP alignment significantly predicted affective ratings ($\beta = 0.176$, $t = 5.12$) and moderated the instruction effect (*condition* $\times$ *alignment*: $\beta = -0.242$, $t = -4.79$). An additional interaction between image type and alignment ($\beta = -0.140$, $t = -2.91$) indicated that alignment effects were stronger for negative than for neutral stimuli. Importantly, the three-way interaction (*image type* $\times$ *condition* $\times$ *alignment*) was significant ($\beta = 0.203$, $t = 2.89$), confirming that the moderating influence of alignment differed across emotional contexts. These findings demonstrate that the observed effects are robust across two independent alignment formulations, mitigating concerns about potential AI-specific bias.
